# Trophic Structure and Isotopic Niche of Invaded Benthic Communities on Tropical Rocky Shores

**DOI:** 10.3390/biology13121023

**Published:** 2024-12-07

**Authors:** Larissa M. Pires-Teixeira, Vinicius Neres-Lima, Plínio C. Barbosa, Joel C. Creed

**Affiliations:** 1 Programa de Pós-Graduação em Ecologia e Evolução, Universidade do Estado do Rio de Janeiro, Rua Francisco Xavier 524, PHLC, Sala 220, Rio de Janeiro 20559-900, RJ, Brazil; 2 Departamento de Ecologia, IBRAG, Universidade do Estado do Rio de Janeiro, Rua Francisco Xavier 524, PHLC, Sala 220, Rio de Janeiro 20559-900, RJ, Brazil; vinicius.lima.eco@gmail.com (V.N.-L.); jcreed@uerj.br (J.C.C.); 3 Centro de Energia Nuclear na Agricultura, Universidade de São Paulo, CENA-USP, Av. Centenário, 303, São Dimas, Piracicaba 13416-000, SP, Brazil; pcamargo@cena.usp.br; 4 Coral-Sol Research, Technological Development and Innovation Network, Instituto Brasileiro de Biodiversidade, Rua Senador Dantas, 20, 1509, Rio de Janeiro 20031-205, RJ, Brazil

**Keywords:** aquatic invasions, non-native species, isotopic niche metrics, suspension feeders, empty niche theory, biotic resistance hypotheses, trophic relationships

## Abstract

When a species is introduced in a new location, it is common for it to establish itself when it finds favorable conditions in the receptor community with regard to interspecific interactions with native species. The azooxanthellate corals *Tubastraea* coccinea and *Tubastraea tagusensis* are invasive species introduced in the Caribbean Sea, the Gulf of Mexico, and the Brazilian Southwest Atlantic. They are successful competitors for space, have multiple reproductive modes, and have high larval dispersion and recruitment, but studies on food and trophic relationships of the genus *Tubastraea* are still scarce. In the present study, we used isotopic values of δ^13^C and δ^15^N to investigate trophic relationships in rocky shore communities invaded by *T. tagusensis* and *T. coccinea* corals under different oceanographic and anthropogenic contexts. Using metrics derived from the isotopic values, we show that invaded communities have a lower degree of trophic diversity, with species characterized by similar trophic ecologies while abiotic factors seem to contribute to the biotic resistance of communities exposed to invasion events. *Tubastraea* spp. occupy a niche space similar to that occupied by the native community of suspension feeders, sharing resources already consumed by the receptor community, which makes invading corals successful competitors for food.

## Simple Summary

The introduction of species into a new location is mediated by different factors, including the favorable conditions that the invasive species encounters in the host community. Among the non-native marine species well known for their potential as invasives, the azooxanthellate corals *Tubastraea coccinea* and *Tubastraea tagusensis*, introduced in the Caribbean Sea, the Gulf of Mexico and in the Brazilian Southwest Atlantic, stand out for their successful competition for space, multiple reproductive modes and high larval dispersal and recruitment. However, little is known about the feeding and trophic relationships of species of the genus *Tubastraea*, a gap that we sought to fill in the present study. Here, we show that invaded communities have a lower degree of trophic diversity, with species characterized by similar trophic ecologies. We also show that the invasive coral *Tubastraea* spp. occupies a trophic niche, similar to that of native species that are suspension feeders. Our results suggest that *Tubastraea* spp. is also a successful invasive species due to its superior competition for food.

## 1. Introduction

Marine and coastal ecosystems around the world are being invaded at extraordinary rates as a result of human transport of non-native species (NS) but the amount of effort dedicated to the study of invasive species in these ecosystems is still inconsistent [1,2,3]. The introduction of an NS can change the diversity in the invaded community and modify interactions between species, leading to the decrease and extinction of native species, disruption of ecosystem functions, and substantial damage to natural resources and ecosystem services [4,5].

For an NS to establish itself, where it is introduced, it needs to be able to extract enough food resources from the environment to support the continuous mass and energy demands associated with growth, survival, and reproduction, so understanding NS diets helps to identify physiological characteristics that contribute to or limit the success of the invasion [6]. The introduction of species into aquatic ecosystems can profoundly disrupt the strong trophic links existing in those communities, making these environments ideal ecosystem models to test hypotheses about the direct and indirect ecological impacts of NS [7]. In the invaded area, the effects on the trophic web will depend on the invader’s trophic position, feeding strategy, and its ability to modify the habitat [7,8,9]. Omnivorous consumers, such as suspension or filter-feeding organisms, can prey (and compete) at more than one trophic level, controlling, for example, the abundance of phytoplankton and zooplankton or larval supply. Predators with rapid growth capacity can reduce the abundance and biomass of important food resources and thus their consumers. NS that are considered ecosystem engineers can alter primary production and nutrient cycles, creating novel habitats and modifying the abundance of organisms in the invaded area, as well as altering other essential ecosystem processes [7,10].

In coastal marine ecosystems, biological invasions can considerably increase the number of suspension feeders, detritivores, deposit feeders, and other primary consumers, when adding a new component to the trophic web [11]. In addition, NS are often generalists that may access unexploited resources thus occupying a vacant trophic niche or reducing niche overlap with populations from the native community [12,13]. Knowledge of the trophic interactions in an invaded community can help to understand and assess the impacts on the trophic structure and composition of the community and even better manage invasion events [13,14,15,16,17].

Stable isotope analysis is a tool used to characterize the trophic structure of an ecosystem. There are other methodologies adopted in food web studies, such as the analysis of stomach contents; however, the use of isotopes provides us with more integrated information about what the organism assimilates, and not only what the animal has recently ingested [18,19], which may or may not be digested and contribute to the consumer’s nutrition [19]. Isotopic values, especially carbon (δ^13^C) and nitrogen (δ^15^N), are effective natural tracers for monitoring energy and nutrient flows, estimating trophic levels, resource use, and the composition of consumers’ diets [20,21,22]. These two stable isotopes are the most often used to assess trophic interactions, as they make it possible to identify different sources of basal food resources and to estimate the trophic position of consumers, respectively [23,24,25]. The use of δ^13^C × δ^15^N biplots makes it possible to infer aspects of the studied species’ trophic niche in the food web from its relative position in the isotopic space [26,27]. Although this is a well-established procedure for studying the trophic structure and dynamics of communities, there are alternative analytical approaches that allow the calculation of descriptors of the trophic structure of a community using stable isotope ratios which, depending on the hypotheses or questions addressed in the research and the general methodological approach of the study, is a promising tool [28,29,30,31,32]. One example is the population and community isotopic niche structure metrics that reflect specific aspects of trophic structure such as width and niche overlap, providing an understanding of its trophic ecology by reflecting some aspects of its trophic niche [33,34,35]. Some gaps still need to be filled, especially in studies of rocky shores and coral reefs, including the comparison of feeding strategies across guilds, and the analysis of multiple taxonomic groups in situ or multiple environmental drivers [36]. Additionally, although widely used to investigate the consumptive effects of non-native predators, in invaded rocky shores, analyses of δ^13^C and δ^15^N of invasive corals as consumers or as a resource to be consumed are rarely used [6,37,38].

When a species is introduced in a new location, it is common for it to establish itself when it finds favorable conditions in the receptor community (i.e., niche requirements) [12], bringing about novel interspecific interactions with native species (i.e., competition and predation) [7]. If the NS is a predator, it may cause direct “top-down effects” on its prey with cascading consequences for lower trophic levels; or if as well as being a predator, it is also a resource for predators at higher trophic levels; it can cause “bottom-up effects” on the food chain by acting as a new food source. A predatory NS may also compete with native species for resources [6]. In studies of invaded communities, values and, metrics derived from stable isotope data are particularly interesting tools for studying the impact of invasions, as they are a way of analyzing the ecosystem based on trophic relationships between species [39]. Biplots with stable isotope values can provide evidence of interactions between populations of native species and NS, interactions between two NS, compare invaded and uninvaded areas, and observe trophic changes following an invasion event. Additionally, metrics can be applied to assess the trophic niche in invaded and uninvaded communities and quantify niche overlap [40,41,42,43]. Stable isotope analyses are therefore a promising tool for predicting the impacts of invaders on native communities and identifying potential management options [38,41,44].

Successful invasive species in the Atlantic, the azooxanthellate corals *Tubastraea coccinea* Lesson, 1830 and *Tubastraea tagusensis* Wells, 1982 were first introduced in the Caribbean Sea by shipping or floating platforms and into the Gulf of Mexico and the Brazilian Southwest Atlantic on oil platforms [45]. In Brazil, *Tubastraea* spp. have already been found on rocky shores and oil platforms, along more than 3000 km of the coast at depths ranging between 1 and 22 m (with a record of a single colony of *T. coccinea* trawled from 100 m depth in the state of São Paulo) [45,46]. The species of the genus *Tubastraea* are heterotrophic and obtain food exclusively through particle-feeding mechanisms (i.e., have no association with symbiotic photosynthetic zooxanthellae). Due to the highly invasive nature and multiple impacts, a large number of studies have now been carried out regarding the expansion, distribution, and increased abundance of *Tubastraea* spp. [45,46,47,48,49,50,51,52], as well as investigations of competition with native species, chemical defenses, reproduction, and facilitation of other species [53,54,55,56,57,58,59,60]. However, studies on feeding and trophic relationships of species of the genus *Tubastraea* are still scarce [37,61,62].

These corals are known to produce bioactive chemicals that deter predators and they are invasive as there are few natural predators amongst the native species [53,54]. This trait may explain the overall dearth of evidence of significant predation of *T. tagusensis* and *T. coccinea* in Brazil and this escape may in part facilitate their highly invasive nature. Studies of the diet of *Tubastraea* spp. in the wild suggest that they may consume heterotrophic prey as well as microalgae when offered in aquaria [63,64,65,66].

Native *Tubastraea* spp. are found in heterogeneous benthic communities in both more pristine locations as well as those suffering from anthropogenic stressors, such as contaminant exposure, benthic damage, abrasion, and smothering [67,68,69,70]. In the invaded range in Brazil native species, such as the zoanthid *Palythoa caribaeorum*, the corals *Siderastrea stellata* and *Mussismilia hispida*, and the sponges *Desmapsamma anchorata* and *Iotrochota arenos*, appear to provide some biotic resistance and favorably compete for space with the invasive corals [55,58,71,72]. However, there is an information gap regarding competition for food resources between *Tubastraea* spp. and other native species in the invaded range. The little evidence from isotopic analysis of δ^13^C and δ^15^N has shown that *Tubastraea* spp. can share the same food source or a similar mixture of food as the probably commensal bivalve *Leiosolenus aristatus* and assimilate resources available in the water column, such as plankton and particulate organic matter (POM) [37,62].

In the present study, we investigated how trophic relationships vary along three rocky shores invaded by *T. tagusensis* and *T. coccinea*. On each rocky shore, the benthic marine communities were sampled in invaded and uninvaded communities. We used isotopic values of δ^13^C and δ^15^N to compare the isotopic niche of the invasive corals and the native benthic species in invaded and uninvaded communities. We also conducted a review of knowledge of *Tubastraea* spp. as a potential food source (prey) for other species as well as investigated the trophic relationships between potential consumer species and invasive corals using isotopic values of δ^13^C and δ^15^N.

## 2. Materials and Methods

### 2.1. Study Area

**Figure 1:**
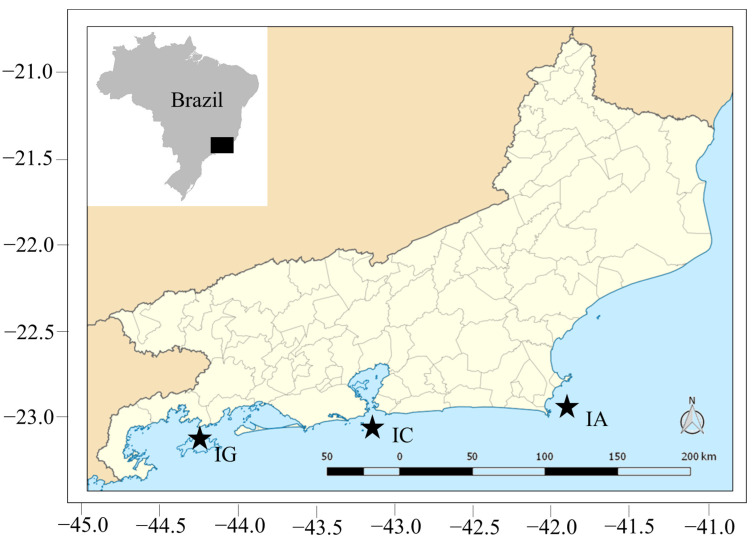
Location of study sites in Rio de Janeiro state, southeast Brazil. IG: Ponta do Bananal, Ilha Grande Bay, Angra dos Reis. IC: Ilha Comprida, Cagarras Archipelago, Rio de Janeiro. IA: Ilha de Âncora, Armação dos Búzios, Cabo Frio region.

Our study was carried out on tropical rocky shores at three distinct locations along the state of Rio de Janeiro, Brazil (Figure 1).

(1) Ponta do Bananal (IG)—located in Ilha Grande Bay, Angra dos Reis, south of the Rio de Janeiro state (23°05′55″ S 44°15′34″ W). It is a region of intense maritime traffic, including shipping and oil platforms in transit. Oil platforms stop for repairs or maintenance or while waiting for berthing at the BrasFels shipyard [47] at the nearby Bananal Anchorage. The IG site is located inside Ilha Grande Bay, which is characterized by shallow, clear water, and was probably the first point of introduction of *Tubastraea* spp. in natural ecosystems in Brazil, by the late 1990s [45].

(2) Ilha Comprida (IC)—located about 5 km from Rio de Janeiro (23°02′15″ S, 43°12′17″ W). It is one of the five islands and two islets that make up the Cagarras Archipelago. This site is on the inside of the archipelago where it is relatively wave protected and has a maximum depth is 40 m. The Cagarras Archipelago is impacted by eutrophic waters from Guanabara Bay (Rio de Janeiro city) [73,74]. *T. tagusensis* was first detected (and removed) from the Cagarras Archipelago in 2004, but in 2011 the species was again present and has expanded at the site [45]. During the period in which the research was carried out, there were no records for *T. coccinea* at this site. Presently, this species is also found, although not very common [75,76].

(3) Ilha de Âncora (IA)—an island located 8 km off the northwest coast of the state of Rio de Janeiro, at Armação dos Búzios, Cabo Frio region (22°46′16″ S, 41°47′08″ W). It is a popular diving spot, with clear, calm shallow waters (depth < 22 m). IA is subjected to seasonal upwelling of nutrient-rich, cold, high salinity South-Central Atlantic Water. It was invaded by *Tubastraea* spp. for the first time in 2011, when the first record was made [45].

### 2.2. Field Sampling and Abiotic Data Measurement

Fieldwork was carried out during the winter, although on different dates, in July 2017 (IG), September 2017 (IC), and June 2018 (IA). Up to five replicate individuals/colonies of each of the most abundant species observed in each community were sampled for analysis of C and N stable isotopes. At sites IA and IC, the samples were taken over 30 m of invaded and uninvaded extensions of the reef. At the IG site, it was not possible to find areas without *Tubastraea* spp. so there we only sampled invaded areas. The areas were determined along a transect stretched parallel along the rocky shore, and collections were made between 3 and 10 m depth in IA and IG, and 5 and 18 m depth in IC. All collections were performed through scuba diving, except for plankton. Due to their size (<1 cm) planktonic organisms and the ophiuroid *O. mirabilis*, were collected in greater quantities to ensure sufficient weight for isotopic analyses to be performed. The encrusting benthic species were removed with a hammer and chisel (corals and bryozoans), scissors (sponges and algae), and spatulas (ascidians and anemones). The turf-forming algal species (a mixture of small entangled filamentous species) were separated after collection for individual analysis. Planktonic organisms were collected with 68 µm nylon plankton nets, in horizontal sampling, for 5 min, to ensure that a quantity was collected that would allow the ideal weight for isotopic analysis. All samples were frozen immediately after collection and taken for screening and preparation in the laboratory. To characterize the conditions on collection we measured temperature, pH, salinity, and dissolved oxygen with a waterproof multi-parameter probe (HI 9828, Hanna Instruments, Woonsocket, RI, USA). Water transparency was estimated as the Secchi depth using a Secchi disk.

### 2.3. Sample Processing

After defrosting the samples, the muscle tissue of the macroconsumers (sea urchins, crustaceans, gastropods, and fish) was collected. Corals and bryozoans had their tissues scraped, while very small animals, such as ophiuroids, were kept whole and individuals grouped together to make up a sample. Soft tissues were sampled for sponges, ascidians, and anemones. All samples were washed with distilled water, so that any contaminants that could influence the isotopic compositions were removed. We removed any epiphytes or epizoa from the animals and macroalgae sampled. The samples were dried in an oven for 48 h at 60 °C and macerated to a fine powder. Organisms with calcium carbonate structures that could not be removed, such as calcareous macroalgae, phytoplankton, zooplankton, bryozoans, hydrozoans, corals, polychaetes and small crustaceans were acidified (only for δ^13^C) [77]. The samples were then placed in tin capsules, weighed, and analyzed for the isotopic composition of carbon and nitrogen at the Centro de Energia Nuclear na Agricultura da Escola Superior de Agricultura Luiz de Queiroz, São Paulo University (CENA-USP).

Stable isotope analyses were performed using a Delta Plus Continuous Flow Isotope Ratio Mass Spectrometer (CF-IRMS, Finnigan MAT, Bremen, Germany) coupled to an elemental analyzer (CE Instruments, Wigan, UK). The isotopic composition is expressed in terms of a delta value (δ) in parts per thousand (‰), obtained by dividing the sample’s isotopic ratio by the isotopic ratio of an internationally accepted standard multiplied by one thousand, according to the formula: δX = [(R_sample_/R_standard_) − 1] × 10^3^, where δX is δ^13^C or δ^15^N and R the ratio ^13^C:^12^C or ^15^N:^14^N. The standard material for carbon was Pee Dee Belamite limestone (PDB) and the standard material for nitrogen was atmospheric air [78]. The standard deviation of isotopic measurements was estimated at 0.09 for δ^13^C and 0.21 for δ^15^N through repeated measures of the internal standard (sugar cane).

### 2.4. Data Analysis

To review the knowledge of *Tubastraea* spp. as a potential food source (prey) for other species, we conducted a literature search during July–November 2024. The searches were performed in the Web of Science database with the keywords ALL = (*Tubastraea*) And Topic = (food OR prey OR source OR resource), which returned only 28 articles. Additionally, manual cover-to-cover searches (“hand searches”) were conducted on Google Scholar, Researchgate, and Scientific Electronic Library Online. Finally, relevant studies known prior to the review were included (Prisma flow diagram: Supplementary File S1). To provide an overview of the trophic structure and identify possible trophic relationships between food sources and consumers, we used a biplot of the values of δ^13^C and δ^15^N of the basal resources and consumers of the invaded sites with the presence of potential predators. We used the SIBER (Bayesian Ellipses to Stable Isotope Data) package [42] in Program R [79] to analyze stable isotope data at the trophic group and species level.

We calculated the isotopic niche metrics in invaded and noninvaded communities in the three studied locations (IG, IC, and IA) using a Bayesian approach to fit multivariate normal distributions to stable isotope data [42]. These distributions can then be used to calculate probability distributions of standard ellipse areas to compare sets of data or to calculate Layman’s metrics to compare entire communities [42]. Layman’s metrics relate the characteristics of the isotopic space filled by each species and reflect important aspects of the trophic structure and trophic diversity of the entire community [33]: (1) N_range (distance between the two species with the maximum and minimum values of δ^15^N, larger intervals suggesting more trophic levels and a greater trophic length); (2) C_range (distance between the two species with the maximum and minimum values of δ^13^C, longer intervals are expected in communities with a greater diversity of basal resources); (3) TA (total isotopic niche area, represents a measure of the total amount of niche space occupied and, therefore, a proxy for niche width); (4) CD (average distance from the centroid, greater distances suggest a higher average degree of trophic diversity within the food chain); (5) MNND (average distance from the nearest neighbor, low values suggesting trophic webs with species characterized by similar trophic ecologies and high trophic redundancy); and (6) SDNND (standard deviation of the distance from the nearest neighbor, low values suggesting a more uniform distribution of the trophic niche). We compared the entire communities invaded versus uninvaded communities at sites IC and IA, using the corresponding distribution of Layman metrics estimated from SIBER (4000 iteration results for each metric) [42]. We also statistically tested the difference between the two groups of communities, invaded and uninvaded, using a *t*-test. With a sample size of 4000, we had a lot of statistical power to detect even small effects (e.g., mean differences) as statistically significant. Consequently, we also had to look at the magnitude of the effects, i.e., the estimated difference between the metrics in invaded and uninvaded communities, to judge whether this is too far from zero to be relevant.

We also performed a more specific analysis among consumers within the same functional feeding characteristics as that of *Tubastraea* spp., that is, suspension-feeding consumers. We used the SEA metric (value of the Standard Ellipse Area that covers 65% of data and indicates the central average of the community’s isotopic niche) [42]. Due to the lack of natural predators, we did not carry out the same analyses for the guild of potential predators of *Tubastraea* spp. [52,60]. To estimate and compare the width of the isotopic niche of *Tubastraea* spp. in the three studied locations (IG, IC, and IA), we used the isotopic niche width analysis measured as the ellipse area (SEAc). A corrected SEA value proposed by Jackson and Parnell (2011) [42] was used to circumvent the bias that arises when sample sizes are small. To determine what fraction of the isotopic niche of suspension feeders was occupied by *Tubastraea* spp., we calculated the overlap of the SEAc of *Tubastrea* spp. and the SEAc of the suspension feeder guild and divided the overlapped area by the SEAc of this guild in each invaded community.

## 3. Results

### 3.1. Tubastraea spp. as a Potential Food Source

We found twelve studies reporting twelve different consumption interactions of *Tubastraea* spp. by generalist and specialist predators, and one personal observation. Among them, only two reports are from places where *Tubastraea* spp. is invasive and most of the interactions in the native regions were with highly specialized predators (Table 1).

### 3.2. Abiotic Characterization

Measurements of the abiotic parameters of seawater confirmed the overall general oceanographic characteristics of the sites as described above. The IA site had clearer, less saline, warmer oxygen-rich waters; IG and IC had cooler, more saline waters and turbid waters with lower dissolved oxygen levels (Table 2).

### 3.3. Trophic Characterization of Invaded and Uninvaded Areas

A total of 61 species/taxa were sampled, 16 taxa in invaded and 18 in uninvaded communities at IC, 24 in invaded and 22 in uninvaded at IA, and 26 in the (totally invaded) community at IG were analyzed for δ^13^C and δ^15^N (Supplementary File S2). The consumers with the highest mean value of δ^15^N were the carnivorous fish *Haemulon auroline*, *H. steindachneri*, the omnivorous fish *Stephanolepis hispidus*, and the omnivorous polychaete fireworm *Hermodice carunculata*, all sampled at the IG site invaded by *Tubastraea* spp. The hydroids *Macrorhynchia philippina*, *Millepora alcicornis* and the ascidian *Diplosoma listerianum* were the consumers with the lowest values of δ^15^N, all suspension feeders and all sampled at the IA site in invaded (*M. philippina* and *D. listerianum*) and uninvaded (*M. alcicornis*) communities. The mean values of δ^15^N of the basal food resources varied between 5.58‰ for the articulated coralline algae *Jania adhaerens* (Rhodophyta) and 8.23‰ for the unidentified crustose coralline algae (Rhodophyta), both at IG in the invaded communities. The macroalgae showed average values of δ^13^C, ranging from −6.17‰ in *J. adhaerens* (invaded community—IA) to −20.35‰ and −20.31‰ in *Hypnea* sp. and *J. adhaerens*, respectively (both at IA in not invaded communities).

**Figure 2:**
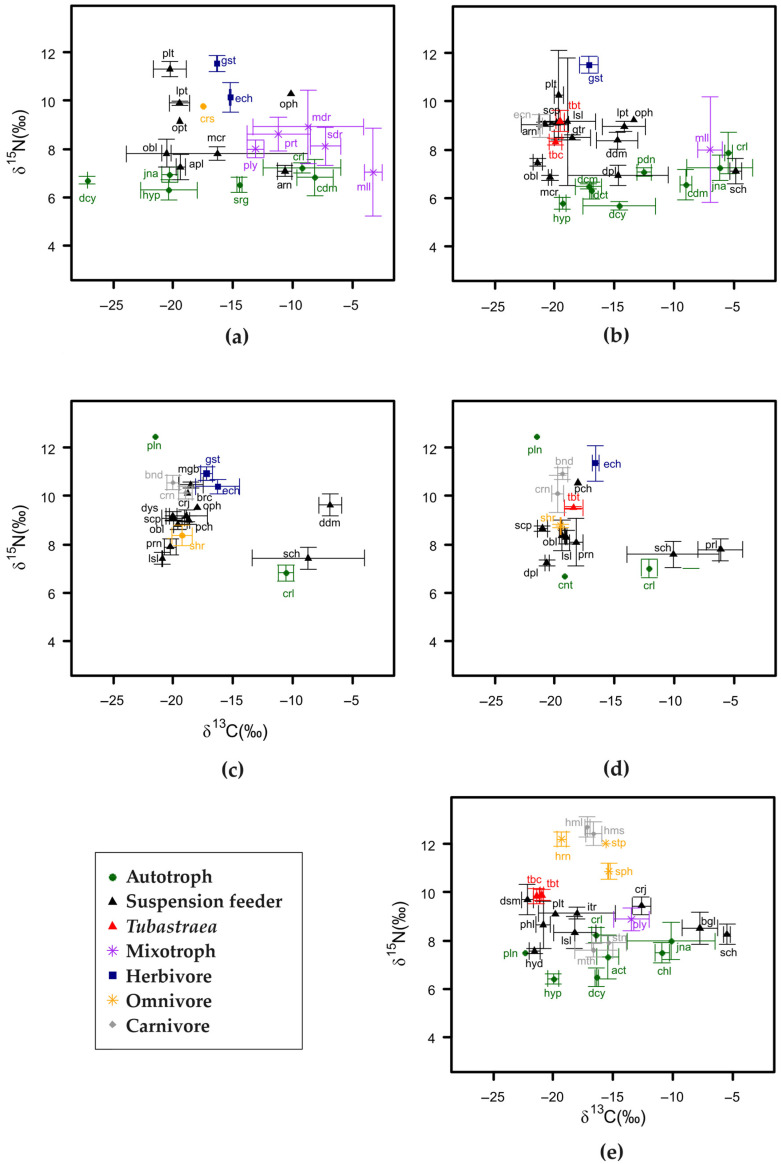
Biplot of stable isotopes of common species in invaded and uninvaded communities on tropical rocky reefs at three studied locations in the southwest Atlantic, Brazil: Ilha de Âncora—IA (not invaded: (**a**); invaded: (**b**)), Ilha Comprida—IC (uninvaded: (**c**); invaded: (**d**)), and Ponta do Bananal—IG (**e**). Symbols indicate the average values (±SD) of resources and consumers separated by guilds. Code by guild: Autotroph (in green): act—*Acetabularia schenckii*, cdm—*Codium intertextum*, chl—*Chlorophyta*, cnt—*Centroceras* sp., crl—*Crustose coralline* algae, dcm—*Dictyota menstrualis*, dct—*Dictyopteris* sp., dcy—*Dictyota* sp., hyp—*Hypnea* sp., jna—*Jania adhaerens*, pdn—*Padina gymnospora*, pln—plankton, srg—*Sargassum vulgare*. Suspension feeders (in black, with the exception of *Tubastraea* spp. corals which are in red): apl—*Aplysina fulva*, arn—*Arenosclera brasiliensis*, bgl—*Bugula* sp., brc—*Brachidontes solisianus*, crj—*Carijoa riisei*, ddm—*Didemnum perlucidum*, dpl—*Diplosoma listerianum*, dsm—*Desmapsamma anchorata*, dys—*Dysidea etheria*, gtr—*Guitarra sepia*, hyd—hydrozoan, itr—*Iotrochota birotulata*, lpt—*Leptogorgia punicea*, lsl—*Leiosolemus aristatus*, mcr—*Macrorhynchia philippina*, mgb—*Megabalanus coccopoma*, obl—*Obelia dichotoma*, oph—Ofiuroidea, opt—*Ophiothela mirabilis*, pch—*Pachycheles monilifer*, phl—*Phalusia nigra*, plt—Tubular polychaete, prl—*Paraleucilla magna*, prn—*Perna perna*, sch—*Schizoporella unicornis*, scp—*Scopalina ruetzleri*, tbc—*Tubastraea coccinea*, tbt—*Tubastraea tagusensis*. Mixotroph (in purple): mdr—*Madracis decatis*, mll—*Millepora alcicornis*, ply—*Paliythoa caribaeorum*, prt—*Porites branneri*, sdr—*Siderastrea stellate*. Herbivore (in blue): ech—*Echinometra lacunter*, gst—Gastropod. Omnivore (in yellow): crs—Crustacea, hrn—*Hermodice carunculata*, shr—Shrimp, sph—*Sphoeroides spengleri*, stp—*Stephanolepis hispidus*. Carnivore (in gray): crn—*Cronius ruber*, hml—*Haemulon auroline*, hms—*Haemulon steindachneri*, bnd—*Bunodosoma caissarum*, ecn—*Echinaster brasiliensis*, mth—*Mithraculus forceps*, stn—*Stenorhynchus seticornis*.

There was little intrageneric variation and the δ^13^C values of *T. coccinea* and *T. tagusensis* were grouped, as were the δ^15^N values. IG was the only site where it was possible to sample a potential predator, the omnivorous fireworm *Hermodice carunculata* (which the literature search revealed had been previously observed to feed on *Tubastraea* spp. in Brazil), and it had isotopic values consistent with the consumption of the invasive corals (Figure 2e). At IA, no omnivorous or carnivorous consumers had isotopic values of δ^13^C and δ^15^N that were consistent with predation on *Tubastraea* spp. (Figure 2b). At IC, a single omnivorous predator, the red swimming crab *Cronius ruber*, presented values consistent with it being a potential consumer of the invading corals (Figure 2d).

Suspension-feeding consumers showed isotopic values similar to those of *Tubastraea* spp., inlcuding the sponge *Desmapsamma anchorata* at IG, the sponges *Guitarra sepia*, *Arenosclera brasiliensis*, and *Scopalina ruetzleri* at IA, and an unidentified tubicolous polychaeta worm at IA (Figure 2b). At the IC site, no consumers had δ^13^C and δ^15^N values overlapping those of *T. tagusensis*, and the consumers with the closest values were the brown mussel *Perna perna*, which is also a suspension (filter) feeder (Figure 2d).

The isotopic values of plankton at IG and the macroalga *Hypnea* sp. at IG and IA are consistent with their consumption by invading corals *Tubastraea* spp. and the above-mentioned suspension feeding (Figure 2b,e). The red macroalga *Centroceras* sp. was another resource which had values consistent with its consumption by *T. tagusensis* at IC, although only one sample was analyzed (Figure 2d).

### 3.4. Isotopic Niche Analyzes

**Figure 3:**
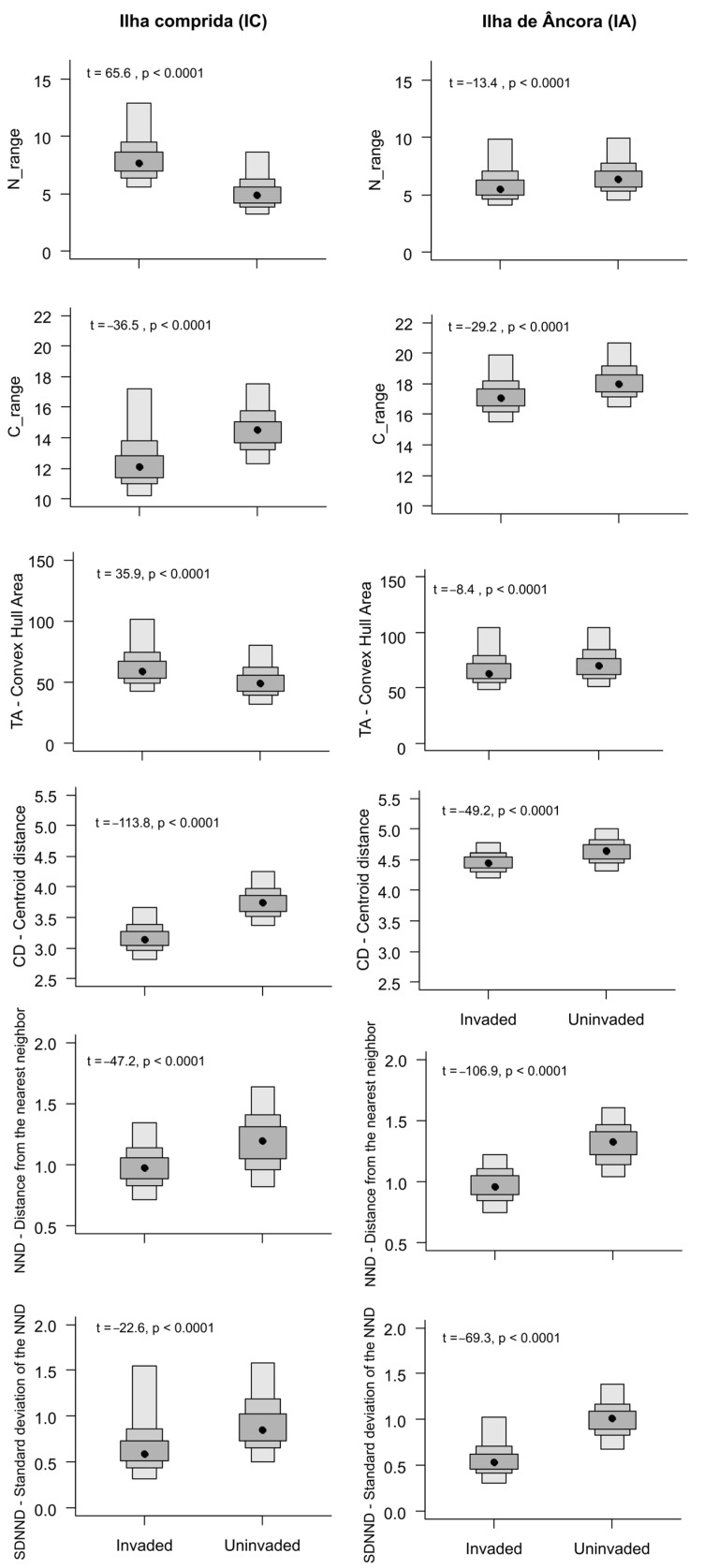
Bayesian posterior distribution of the 6 Layman metrics of invaded and uninvaded areas and *t*-test results. The box edges represent 95, 75, and 50% credible intervals and the point in the middle of the boxes represents the mode.

The total niche area (TA) was greater in the community uninvaded at IA (uninvaded: 76.32; invaded: 58.64) and was very similar between invaded (41.32) and uninvaded communities at IC (41.85). The C_range in IA was higher in the uninvaded community (uninvaded: 23.94; invaded: 16.56). Also, at IA, the SDNND in the uninvaded community was more than double that of the invaded community (uninvaded: 1.34; invaded: 0.64). The other metrics showed similar values, regardless of location or status (invaded or uninvaded). All metrics showed significant differences between invaded and uninvaded areas (Figure 3).

**Figure 4:**
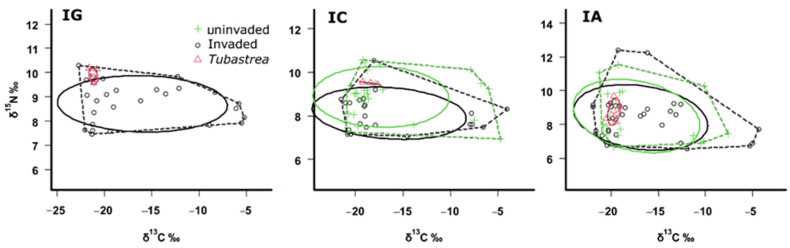
The isotopic niche of suspension-feeding consumers in uninvaded (green) and invaded (black) benthic communities on tropical rocky reefs at three studied locations along the coast of the state of Rio de Janeiro in the southwest Atlantic, Brazil. IG: Ponta do Bananal; IC: Ilha Comprida; IA: Ilha de Âncora. Dashed line—convex hulls area; continuous line—ellipse areas with a 65% confidence interval.

The guild of suspension feeders occupies a wide area of isotopic niche in both invaded and uninvaded communities (in the invaded area: at IG, SEA= 15.62; IA, SEA = 20.10; at IC, SEA= 14.28; in the uninvaded area: at IA, SEA = 21.5; at IC, SEA= 14.87) (Figure 4). In invaded communities, the average values of δ^13^C of suspension feeders range from −22.73‰ (*D. anchorata* at IG) to −5.16‰ (*Schizoporella unicornis* at IG), and δ^15^N ranged from 5.87‰ (*Millepora alcicornis* at IA) to 12.38‰ (unidentified tubicolous polychaete worm at IA). In uninvaded communities, the average values of δ^13^C of suspension feeders range from −20.91‰ (*Leiosolemus aristatus* at IC) to −3.25‰ (*M. alcicornis* at IA), and δ^15^N ranged from 7.04‰ (*M. alcicornis* at IA) to 11.54‰ (gastropod at IA). The niche area, represented by SEAc, of *Tubastraea* spp. occupied a specific and limited area of the standard ellipse of suspension feeders in the three locations (at IG: 1.76%; at IA: 4.63%; at IC: 0.02%) (Figure 4).

## 4. Discussion

This study gives us new insights into trophic relationships in shallow tropical rocky reef communities in the southwest Atlantic which have been invaded by corals *T. tagusensis* and *T. coccinea*. These insights include an assessment of interactions between the two invasive species and native counterparts, as well as the first information on trophic relationships involving predation and competition for food resources by species of the genus *Tubastraea* within their invasive range. To do so, we used the isotopic values of δ^13^C and δ^15^N in three different invaded and uninvaded communities with differing oceanographic and anthropogenic settings.

Regarding an overview of the trophic structure of the sampled sites, the nitrogen isotopic values reflected the trophic position of the species, with the basal food resources, represented by macroalgae from three different phyla, presenting the lowest δ^15^N values, when compared to the values of consumers, higher for carnivores and omnivores [89,90,91]. In addition to the macroalgae, the mixotrophic hydrocoral *Millepora alcicornis* (a consumer with symbiotic zooxanthellae algae) represented an additional source of resources reflected in lower values of δ^15^N assimilation. The δ^13^C values of the basal food resources were notably wide, especially in the Rhodophyta. The wide variation in δ^13^C values of marine algae, which can vary from –3‰ to –35‰, has previously been documented in an extensive review [92]. Macroalgae are exposed to a range of water movement that varies according to depth, tidal stage, wave action, and currents; such variation coupled with the polyphyletic nature or algae and their consequent diverse metabolic pathways have effects on inorganic carbon and other nutrients available for assimilation [92,93]. However, while others have attributed variability in values to taxonomy and the ecology of the macroalgae studied, we found considerable variation in δ^13^C within the same species at the same collection site within invaded and uninvaded areas (e.g., *Jania adhaerens* at IA: −20.31‰ in the uninvaded area and −6.17‰ in the invaded area). Also, in our study, not only Rhodophyta, but Phaeophyta like *Dictyota* sp. (in IA uninvaded: −27.18‰) and *Padina gymnospora* (in IA invaded: −12.51‰) also showed variation and our collections were carried out between 5 and 10 m in depth, differently from other locations where Rhodophyta with δ^13^C values below –30‰ were from submarine, shaded or intertidal environments [93]. Finally, most of the red algae (from the North-East Atlantic) that had very low δ^13^C values were collected under low light and temperatures [92], but in the locations of our study, the lowest temperature recorded was at IC (18.39 °C). We suggest that future studies assess the variation in δ^13^C in benthic marine algae over a wider latitudinal range or from a phylogenic perspective.

The sponges *D. anchorata*, *G. sepia*, *A. brasiliensis*, and *S. ruetzleri* presented values of δ^13^C and δ^15^N close to the values of *Tubastraea* spp. Other studies have shown that, in the invaded range in Brazil, the encrusting sponge *S. ruetzleri* is among the Porifera that most frequently compete for space by contact with *Tubastraea* spp., and *D. anchorata* is one of the few benthic species capable of overgrowing and occasionally killing the invasive corals [72,94]. The similar isotopic values of these consumers and the evidence of interaction already reported in the literature suggest some degree of competition between them, such as sharing similar diets, engaging in close feeding interactions, or taking advantage of similar resources. Future studies investigating the resources that are part of the diet of these organisms may provide further answers about these trophic relationships.

No previous studies have looked at the feeding preferences of corals of the genus *Tubastraea*. As they are suspension-feeding filter-feeding species that feed on resources available in the water column, the constituents of particulate organic matter (POM) are food sources for *Tubastraea* spp. Both *Hypnea* sp. and *Centroceras* sp. are turf-forming red algae that presented δ^13^C and δ^15^N values that suggest a trophic position of a potential resource consumed by *Tubastraea* spp., probably captured as fragments of suspended debris after breaking by waves or herbivores. *Hypnea* sp. is a fast-growing macroalgae that develops as an epiphyte or on primary substrate, contains a considerable amount of protein, and has no chemical protection against predation [95,96]. Studies show that *Hypnea* sp. is an important food resource consumed by fish, sea turtles, crustaceans, and gastropods in shallow marine habitats [95,96,97,98,99]. Similarly, *Centroceras* sp. is a filamentous turf that grows on primary or epiphyte substrate and is consumed by fish, gastropods, and sea urchins [98,100]. Algal turfs are very important for primary productivity in tropical reef environments and are amongst the most abundant taxons/functional groups at our study sites [73]; they have also been found to be the group most excluded by *Tubastraea* spp. through competition [73,101], so again field observations support the interaction with trophic relationships determined from δ^13^C and δ^15^N values. These data also suggest that the mechanism of capture and consumption of suspended food is selective and not simply a function of the composition of particles of food that is presented to the sessile consumer by the currents, in this case, the invasive corals *Tubastraea* spp.

Our results also support another trophic interaction by providing evidence of *Tubastraea* spp. being preyed upon by the fireworm *H. carunculata* [60]. However, it appears that this predation pressure is not strong enough to have a significant impact on invasive *Tubastraea* spp., as they have rapidly expanded throughout the tropical and subtropical southwest Atlantic, and significant damage to polyps that would be conducive to widespread feeding is not observed. These fireworms are not very abundant so damage from predation is infrequent; *Tubastraea* spp. have substantial ability to recover from partial polyp or colony damage [102,103]. One caveat was the number of individuals sampled: even in three equidistant locations, along the coast of Rio de Janeiro, only three individuals of *H. carunculata* were found and sampled in just one location.

Abundant native predators or strong competitors which limit establishment or expansion (population size) or habitat use by NS, and predation can also mediate competitive interactions and, in turn, affect species abundance and structure in the community [104,105,106]. However, strong biotic resistance may occur only when native predators possess characteristics, such as high abundance, strong predation pressure on NS prey, and high feeding rates [107]. These characteristics seem far from the only observed and documented predation of *Tubastraea* spp. in Brazil—three *T. tagusensis* polyps eaten by a fireworm *Hermodice carunculata* previously reported [60], and corroborated by our results. In 20 years of field studies, the starfish *Oreaster reticulatus* has been observed only once preying on *Tubastraea* spp. [author’s (JCC) personal observation]. We did not find individuals of the starfish *O. reticulatus* to be sampled. The carnivorous crustacean *C. ruber* was yet another predator with values of δ^13^C and δ^15^N that were consistent with a possible consumption of *Tubastraea* spp., but it was only possible to sample one individual. In the Indo-Pacific, the native range of *T. coccinea* and *T. micranthus*, these corals are preyed upon by highly specialist predators which are not very voracious consumers but can resist the secondary metabolites used as chemical defense and, in some cases, actually sequester them for their own defense [67,83].

The metrics used to describe important aspects of invaded and non-invaded communities were different and the total extent of trophic diversity within the food webs was greater in the non-invaded communities. Furthermore, the total carbon band exploited by the community reflected a greater abundance of resources exploited by local consumers in the invaded area [14,33], suggesting that invaded communities present a lower degree of trophic diversity, with species characterized by similar trophic ecologies. The three study sites are well-known diving spots, especially for their species diversity. IG and IC are located within Brazilian Conservation Units, that is, priority areas for conservation, sustainable use, and shared benefits of Brazilian biodiversity. IG is located within the Tamoios Environmental Protection Area, an environmental protection area in the state of Rio de Janeiro that protects an area of coastal forests, mangroves, rocky coasts, and islands. IC is part of the Cagarras Islands Archipelago Natural Monument, a federal Conservation Unit of full protection that comprises a group of seven islands and rocks. Although IA is not within a Conservation Unit, the distance of ≈5 km offshore, less urbanization in the surrounding area, and the position of more open waters make it an untouched and little frequented location. IA is also located in the Cabo Frio upwelling region, where primary productivity is high when seasonal upwelling occurs in austral spring–summer (September to November–December to February) and winter (June to August) [108,109]. At sites with upwelling, such as IA, the proportion of algal or autochthonous C available in the austral spring–summer is greater than at other sites across the southwest Atlantic without upwelling. This site therefore differs from other study sites in terms of the amount of organic matter that may influence the basal resources available to *Tubastraea* and other suspension-feeding filter-feeders [110]. This study represents the first isotopic niche analysis involving the invasive corals *Tubastraea*, so additional studies evaluating temporal variation and the effects of seasonality on invaded communities are welcome.

Although suspension-feeding species occupy a wide isotopic niche space, invading corals occupy a specific and limited range within that niche. The likelihood that the invasion will be successful is increased if a new species’ niche requirements overlap little or not at all with species already resident in the receiving community [111]. *Tubastraea* spp. occupied a similar trophic niche space to that occupied by some native species, sharing resources already consumed by the receptor community. Our results suggest that food resources may not be limited compared to other resources and demands and that the outcome of coexistence may result from another type of competition, although evidence in the literature suggests that NS generally need certain limiting resources to establish themselves and spread, being more successful in habitats where competition for these resources is reduced [13,41,112]. Another point to be discussed is that the occupation or domination of space, per se, will directly affect the feeding success of sessile suspension feeders in benthic communities, and *Tubastraea* spp. are well known for their arsenal of both allopathic chemical and physical defenses [53,55,113]. We also confirm the only isotopic characterization ever carried out for a species of *Tubastraea* spp., which points to the same source or a similar mixture of food for the invasive corals and two other suspension feeders the bivalves *Leiosolenus aristatus* and *Crassostrea virginica* [37]. In the Pacific, the δ^13^C and δ^15^N values of *Tubastraea* sp. were similar to those found here, ranging from −21.1‰ to −19.9‰ and 8.6‰ to 9.2 ‰, respectively, [66] were also similar to the δ^13^C of *T. coccinea* in the North Atlantic, −20.27‰ [63], ranging from between −21‰ and −20‰ to δ^15^N between 7‰ and 9‰ [65].

The niche occupied by consumers in the invaded areas was similar in the three studied locations. The amplitude of the suspension feeder niche suggests the consumption of different sources of resources; however, individuals of the same species were grouped in different parts of the isotopic space, suggesting that the available resources were selected by some mechanism(s). The isotopic niches of *Tubastraea* spp., consistent in the three studied locations, were tightly grouped and occupied a characteristic range (c. −21 to −19‰ in δ^13^C and c. 8 to 10‰ in δ^15^N), confirming this observation. Sessile benthic suspension feeding consumers use a variety of foraging behaviors, from feeding on particles large enough to be seized individually to processing the surrounding water (filtering or capturing in mucus nets) for particles so small that they may only be obtained by these methods [114,115]. Ecological filtration functions performed by consumer suspension-feeding species have previously been shown to be different in bivalve species that may select specific components of the suspended particulate material available in aquatic ecosystems [112]. On the other hand, no selectivity has been observed, regarding the size of the prey by species of tunicate and bryozoan suspension feeders [116]. The mechanisms used by *Tubastraea* spp. to select the consumed resources are still unknown and we suggest that further studies are needed to examine and evaluate food selection and uptake by these invasive corals. Another aspect that would be enlightening, mainly for the purpose of comparison with our results, would be studies of the niche of *Tubastraea* spp. in its native range and communities.

## 5. Conclusions

Using C and N isotopic analysis and isotopic niche diversity metrics, this study provides the first trophic characterization of the invading corals *Tubastraea tagusensis* and *Tubastraea coccinea*. Our metrics derived from stable isotopes suggest that greater trophic diversity in uninvaded areas appears to be influenced, not only by the community’s resistance to invasion, but also by the abiotic factors involved (such as increased productivity due to local upwelling events). In light of the trophic niche, the low abundance of native predators, and the low pressure of predation on NS prey, we suggest that there is some level of coexistence and substantial niche overlap of natives and invasives. Our results suggest that *Tubastraea* spp. occupy a quite specific niche within the niche space occupied by other functionally equivalent species. They are successful competitors for food resources but are not desirable food items.

## Figures and Tables

**Table 1:** Species of predators that consume *Tubastraea* spp., the natural distribution of predators, where the predation event was observed, and type of feeding.

Predator Species	Consumed Species	Natural Distribution	Observation Site	Feeding Type		Bibliography
				Generalist	Specialist	
*Coralliophila costularis* (Lamarck, 1816)	*T. megacorallita*; *T. diaphana*	Indo-Pacific	Breaker Reef, Hong Kong	x		[80]
*Epidendrium* sp.	*T. coccinea*	Indo-Pacific	Breaker Reef, Hong Kong		x	[80]
*Epidendrium billeeanum* (DuShane & Bratcher, 1965)	*Tubastraea* spp.	Indo-Pacific	Galapagos; Hawaii; Hawaiian Gulf of California; Maldives; Singapore; Philippines		x	[67,81]
*Epidendrium billeeanum* (DuShane & Bratcher, 1965)	*T. coccinea*	Indo-Pacific	Sudanese Red Sea; Nicaragua (Pacific); Galapagos		x	[82,83]
*Hermodice carunculata* (Pallas, 1766)	*T. tagusensis*	Galapagos Archipelago	Brazil	x		[60]
*Hermodice carunculata* (Pallas, 1766)	*T. aurea*	Indo-Pacific	Venezuela	x		[84]
*Hexaplex princeps* (Broderip, 1833)	*Tubastraea* spp.	Galapagos Archipelago, Indo-Pacific	Galapagos Archipelago		x	[67]
*Oreaster reticulatus* (Linnaeus, 1758)	*Tubastraea* spp.	Galapagos Archipelago, Indo-Pacific	Brazil	x		Author’s personal observation (JCC)
*Phestilla melanobrachia* Bergh, 1874	*T. coccinea*	Indo-Pacific	Sudanese Red Sea; Nicaragua (Pacific); Galapagos		x	[85,86]
*Phestilla melanobrachia* Bergh, 1874	*T. micranthus*	Indo-Pacific	Palau; Gulf of Thailand		x	[85,87]
*Phestilla melanobrachia* Bergh, 1874	*T. diaphana*	Indo-Pacific	Hong Kong		x	[85]
*Phestilla melanobrachia* Bergh, 1874	*T. megacorallita*	Indo-Pacific	Hong Kong		x	[80]
*Phestilla melanobrachia* Bergh, 1874	*T. aurea*	Indo-Pacific	Hawaii (in laboratory)		x	[88]

**Table 2:** Mean (SD) of the oceanographic characteristics of sub-surface water measured by sensors in situ at Ponta do Bananal (IG), Ilha Comprida (IC), and Ilha de Âncora (IA). Different letters indicate significant differences (*p* < 0.05) between Locations IA and IC, as calculated by ANOVA. Due to the absence of replication, the IG location was not included in the analysis of variance.

Parameter	IG	IA	IC
Date	July, 2017	September, 2017	June, 2018
n	1	3	3
Visibility (m)	12 (-)	19.3 (1.15) ^a^	5.33 (2.89) ^b^
pH	7.6 (-)	8.1 (0.03) ^a^	8.5 (0.09) ^b^
Temperature (°C)	21.9 (-)	22.4 (0.44) ^a^	19.5 (1.00) ^b^
DO (mg/L)	11.7 (-)	13.0 (3.79) ^a^	5.98 (1.44) ^b^
Salinity (PSU)	37.9 (-)	32.4 (0.21) ^a^	36.0 (0.96) ^b^

## Data Availability

Data are contained within the article or Supplementary Materials.

## Supplementary Materials

The following supporting information can be downloaded at: https://www.mdpi.com/article/10.3390/biology13121023/s1, File S1: Preferred Reporting Items for Systematic reviews and Meta-Analyses (PRISMA) flow diagram used as a protocol in the review process; File S2: mean values of δ ^13^C and δ^15^N and standard deviation (SD) of primary producers and consumers analyzed in this study.
