# Supplementary material for: Trophic Structure and Isotopic Niche of Invaded Benthic Communities on Tropical Rocky Shores
Source: Biology (Basel). 2024 Dec 7;13(12):1023. doi: 10.3390/biology13121023 (PMC11673876; doi:10.3390/biology13121023)
Supplement: Supplementary file 1 [file biology-13-01023-s001.zip › Online Resource 2.pdf]

Online Resource 2: mean values of δ^{13}C and δ^{15}N and standard deviation (SD) of primary producers and consumers analyzed in this study.

Local	Situation	Taxa	Group	n	δ^{15}N		δ^{13}C	
					value/ mean value	SD	value/ mean value	SD
IC	invaded	*Centroceras* sp.	rhodophyta	1	6.68		-19.21	
IC	invaded	coralline algae	rhodophyta	3	7.02	0.38	-12.90	0.68
IC	invaded	zooplankton	crustacea	1	12.47		-21.55	
IC	invaded	*Cronius ruber*	crustacea	2	10.10	0.77	-19.75	0.48
IC	invaded	*Pachycheles monilifer*	crustacea	1	10.54		-18.05	
IC	invaded	shrimp NI	crustacea	2	8.75	0.08	-19.52	0.03
IC	invaded	*Perna perna*	gastropod	3	8.08	0.97	-18.21	0.56
IC	invaded	*Myoforceps aristatus*	gastropod	3	8.30	0.27	-19.02	0.06
IC	invaded	*Bunodosoma caissarum*	anemone	3	10.91	0.25	-19.34	0.43
IC	invaded	*Obelia dichotoma*	hydrozoa	3	8.37	0.64	-19.40	0.23
IC	invaded	*Schizoporella unicornis*	brydrozoa	3	7.59	0.54	-10.80	3.84
IC	invaded	*Diplosoma listerianum*	ascidiacea	3	7.23	0.10	-20.66	0.2
IC	invaded	*Paraleucilla magna*	porifera	3	7.79	0.46	-6.02	1.88
IC	invaded	*Scopalina ruetzleri*	porifera	3	8.64	0.10	-21.03	0.43
IC	invaded	*Tubastraea tagusensis*	cnidaria	3	9.50	0.05	-18.43	0.78
IC	invaded	*Echinometra lacunter*	echinodermata	3	11.35	0.73	-16.59	0.28
IC	not invaded	coralline algae	rhodophyta	3	6.81	0.33	-10.56	0.65
IC	not invaded	*Obelia dichotoma*	hydrozoa	3	8.80	0.17	-19.59	0.36
IC	not invaded	*Schizoporella unicornis*	bryozoa	3	7.43	0.46	-8.70	4.69
IC	not invaded	*Megabalanus coccopoma*	crustacea	3	10.46	0.11	-18.52	1
IC	not invaded	*Pachycheles monilifer*	crustacea	1	10.10		-18.79	
IC	not invaded	*Cronius ruber*	crustacea	2	10.03	0.27	-18.97	0.54
IC	not invaded	shrimp NI	crustacea	3	8.36	0.42	-19.28	0.85
IC	not invaded	gastrophoda NI	gastropod	3	10.92	0.27	-17.21	0.49
IC	not invaded	*Myoforceps aristatus*	gastropod	3	7.42	0.24	-20.91	0.04

IC	not invaded	*Perna perna*	gastropod	3	7.89	0.32	-20.23	0.5
IC	not invaded	*Brachidontes solisianus*	gastropod	3	8.89	0.18	-18.71	0.1
IC	not invaded	*Scopalina ruetzleri*	porifera	3	9.08	0.07	-20.01	0.94
IC	not invaded	*Dysidea etheria*	porifera	3	9.16	0.20	-20.01	0.32
IC	not invaded	*Bunodosoma caissarum*	anemone	3	10.55	0.29	-20.01	0.53
IC	not invaded	*Didemnum perlucidum*	ascidiacea	3	9.62	0.46	-6.86	0.95
IC	not invaded	*Echinometra lacunter*	echinodermata	3	10.38	0.30	-16.26	1.84
IC	not invaded	ofiuroidea NI	echinodermata	1	9.50		-17.98	
IC	not invaded	*Carijoa riisei*	cnidaria	3	9.16	0.25	-18.88	1.72
IA	invaded	*Jania adhaerens*	rhodophyta	3	7.25	0.51	-6.17	2.77
IA	invaded	*Hypnea* sp.	rhodophyta	2	5.78	0.25	-19.35	0.29
IA	invaded	*Codium intertextum*	chlorophyta	3	6.56	0.62	-9.02	0.49
IA	invaded	*Dictyopteris* sp.	phaeophyceae	3	6.31	0.36	-16.92	0.11
IA	invaded	*Padina gymnospora*	phaeophyceae	3	7.06	0.17	-12.51	0.6
IA	invaded	*Dictyota menstrualis*	phaeophyceae	3	6.48	0.11	-17.17	1.12
IA	invaded	*Dictyota sp.*	phaeophyceae	3	5.67	0.18	-14.59	3.04
IA	invaded	*Obelia dichotoma*	hydrozoa	3	7.47	0.15	-21.41	0.4
IA	invaded	*Macrorhynchia philippina*	hydrozoa	2	6.84	0.11	-20.39	0.12
IA	invaded	*Millepora alcicornis*	hydrozoa	3	7.99	2.19	-6.98	1.03
IA	invaded	*Schizoporella unicornis*	bryozoa	3	7.11	0.52	-4.85	0.5
IA	invaded	*Leptogorgia punicea*	gorgoniidae	3	8.96	0.22	-14.18	1.8
IA	invaded	*Tubastraea coccinea*	cnidaria	3	8.32	0.14	-19.92	0.54
IA	invaded	*Tubastraea tagusensis*	cnidaria	3	9.18	0.43	-19.53	0.37
IA	invaded	*Diplosoma listerianum*	ascidiacea	3	6.94	0.41	-14.69	4.18
IA	invaded	*Didemnum perlucidum*	ascidiacea	3	8.38	0.35	-14.75	1.72
IA	invaded	*Scopalina ruetzleri*	porifera	3	9.11	0.06	-18.81	0.59
IA	invaded	*Guitarra sepia*	porifera	3	8.49	0.11	-18.52	1.15
IA	invaded	*Arenosclera brasiliensis*	porifera	3	9.05	0.09	-20.81	1.96
IA	invaded	gastropod NI	gastropod	3	11.51	0.35	-17.12	0.79

IA	invaded	*Myoforceps aristatus*	gastropod	3	9.16	2.65	-18.93	2.37
IA	invaded	tubular polychaete NI	polychaeta	3	10.26	1.84	-19.65	0.4
IA	invaded	ofiuroidea NI	echinodermata	1	9.22		-13.36	
IA	invaded	*Echinaster brasiliensis*	echinodermata	3	8.98	0.48	-21.21	0.2
IA	not invaded	*Jania adhaerens*	rhodophyta	3	6.94	0.32	-20.31	0.67
IA	not invaded	coralline algae	rhodophyta	3	7.23	0.22	-9.22	3.24
IA	not invaded	*Hypnea sp.*	rhodophyta	3	6.30	0.42	-20.35	2.4
IA	not invaded	*Sargassum vulgare*	phaeophyceae	3	6.52	0.32	-14.46	0.19
IA	not invaded	*Dictyota sp.*	phaeophyceae	3	6.70	0.16	-27.18	0.02
IA	not invaded	*Codium intertextum*	chlorophyta	3	6.81	0.77	-8.13	1.51
IA	not invaded	*Obelia dichotoma*	hydrozoa	3	7.81	0.58	-20.56	0.4
IA	not invaded	*Millepora alcicornis*	hydrozoa	3	7.04	1.82	-3.25	0.75
IA	not invaded	*Macrorhynchia philippina*	hydrozoa	3	7.80	0.28	-16.31	7.58
IA	not invaded	*Ophiothela mirabilis*	hydrozoa	1	9.14		-19.44	
IA	not invaded	ofiuroidea NI	echinodermata	1	10.28		-10.12	
IA	not invaded	*Siderastrea stellata*	cnidaria	3	8.11	0.78	-7.26	1.28
IA	not invaded	*Madracis decatis*	cnidaria	3	8.92	1.52	-8.70	4.61
IA	not invaded	*Porites branneri*	cnidaria	3	8.62	0.69	-11.19	2.64
IA	not invaded	*Echinometra lacunter*	echinodermata	3	10.13	0.62	-15.21	0.08
IA	not invaded	*Leptogorgia punicea*	gorgoniidae	3	9.89	0.09	-19.46	0.83
IA	not invaded	*Paliythoa caribaeorum*	zoanthidea	3	7.99	0.36	-13.10	0.69
IA	not invaded	*Arenosclera brasiliensis*	porifera	3	7.08	0.23	-10.64	0.6
IA	not invaded	*Aplysina fulva*	porifera	3	7.25	0.53	-19.37	0.37
IA	not invaded	crustacea NI	crustacea	1	9.78		-17.47	
IA	not invaded	gastropod NI	gastropod	3	11.54	0.35	-16.33	0.06
IA	not invaded	tubular polychaete	polychaeta	2	11.30	0.30	-20.28	1.38
IG	invaded	coralline algae	rhodophyta	3	8.23	0.32	-16.40	0.36
IG	invaded	*Jania adhaerens*	rhodophyta	2	5.58	0.78	-10.14	3.67
IG	invaded	*Hypnea* sp.	rhodophyta	3	6.42	0.21	-19.99	0.48

IG	invaded	Chlorophyta NI	chlorophyta	3	7.49	0.41	-10.91	0.6
IG	invaded	*Acetabularia schenckii*	phaeophyceae	3	7.33	0.90	-15.43	0.91
IG	invaded	*Dyctiota* sp.	phaeophyceae	2	6.47	0.38	-16.37	0.15
IG	invaded	hydrozoa NI	hydrozoa	3	7.56	0.10	-21.56	0.45
IG	invaded	*Schizoporella unicornis*	bryozoa	2	8.43	0.41	-5.46	0.42
IG	invaded	*Bugula* sp.	bryozoa	3	8.51	0.66	-7.73	1.47
IG	invaded	zooplankton	bryozoa	1	7.50		-22.40	
IG	invaded	*Stenorhynchus seticornis*	crustacea	3	7.91	0.31	-15.34	1.45
IG	invaded	*Mithraculus forceps*	crustacea	3	7.60	0.27	-16.61	1.59
IG	invaded	*Myoforceps aristatus*	crustacea	3	8.32	0.66	-18.21	1.75
IG	invaded	*Iotrochota birotulata*	porifera	3	9.13	0.25	-18.01	3.23
IG	invaded	*Desmapsamma anchorata*	porifera	3	9.69	0.63	-22.17	0.49
IG	invaded	*Phalusia nigra*	ascidiacea	3	8.66	0.98	-20.84	0.63
IG	invaded	*Tubastraea coccinea*	cnidaria	3	9.84	0.31	-21.39	0.25
IG	invaded	*Tubastraea tagusensis*	cnidaria	3	9.88	0.23	-20.80	0.16
IG	invaded	*Carijoa riisei*	cnidaria	3	9.43	0.37	-12.63	0.76
IG	invaded	*Paliythoa caribaeorum*	zoanthidea	3	8.88	0.48	-13.46	1.16
IG	invaded	tubular polychaete	polychaeta	1	9.08		-19.80	
IG	invaded	*Hermodice carunculata*	polychaeta	3	12.19	0.30	-19.30	0.38
IG	invaded	*Haemulon steindachneri*	fish	3	12.42	0.50	-16.62	0.66
IG	invaded	*Haemulon auroline*	fish	3	12.70	0.43	-17.15	0.2
IG	invaded	*Stephanolepis hispidus*	fish	1	12.01		-15.59	
IG	invaded	*Sphoeroides spengleri*	fish	2	10.86	0.34	-15.34	0.09
