# Supplementary figures and images for: Trophic Structure and Isotopic Niche of Invaded Benthic Communities on Tropical Rocky Shores
Source: Biology (Basel). 2024 Dec 7;13(12):1023. doi: 10.3390/biology13121023 (PMC11673876; doi:10.3390/biology13121023)

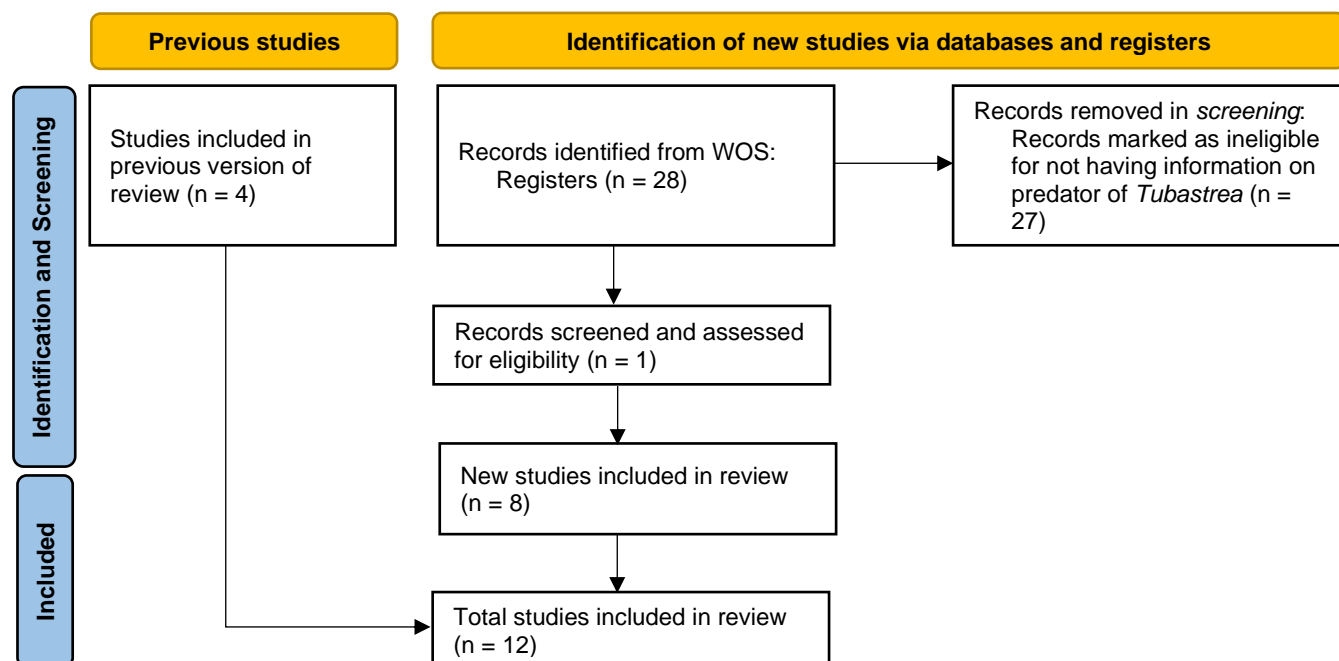

Supplement: Supplementary file 1 [file biology-13-01023-s001.zip › Online Resource 1.pdf]
